# Supplementary material for: An Improved αvβ6-Receptor-Expressing Suspension Cell Line for Foot-and-Mouth Disease Vaccine Production
Source: Viruses. 2022 Mar 16;14(3):621. doi: 10.3390/v14030621 (PMC8951101; doi:10.3390/v14030621)
Supplement: Supplementary file 1 [file viruses-14-00621-s001.zip › SP_BHK-21 manuscript_Supplementary data_V1_JS.pptx]

## Slide 1
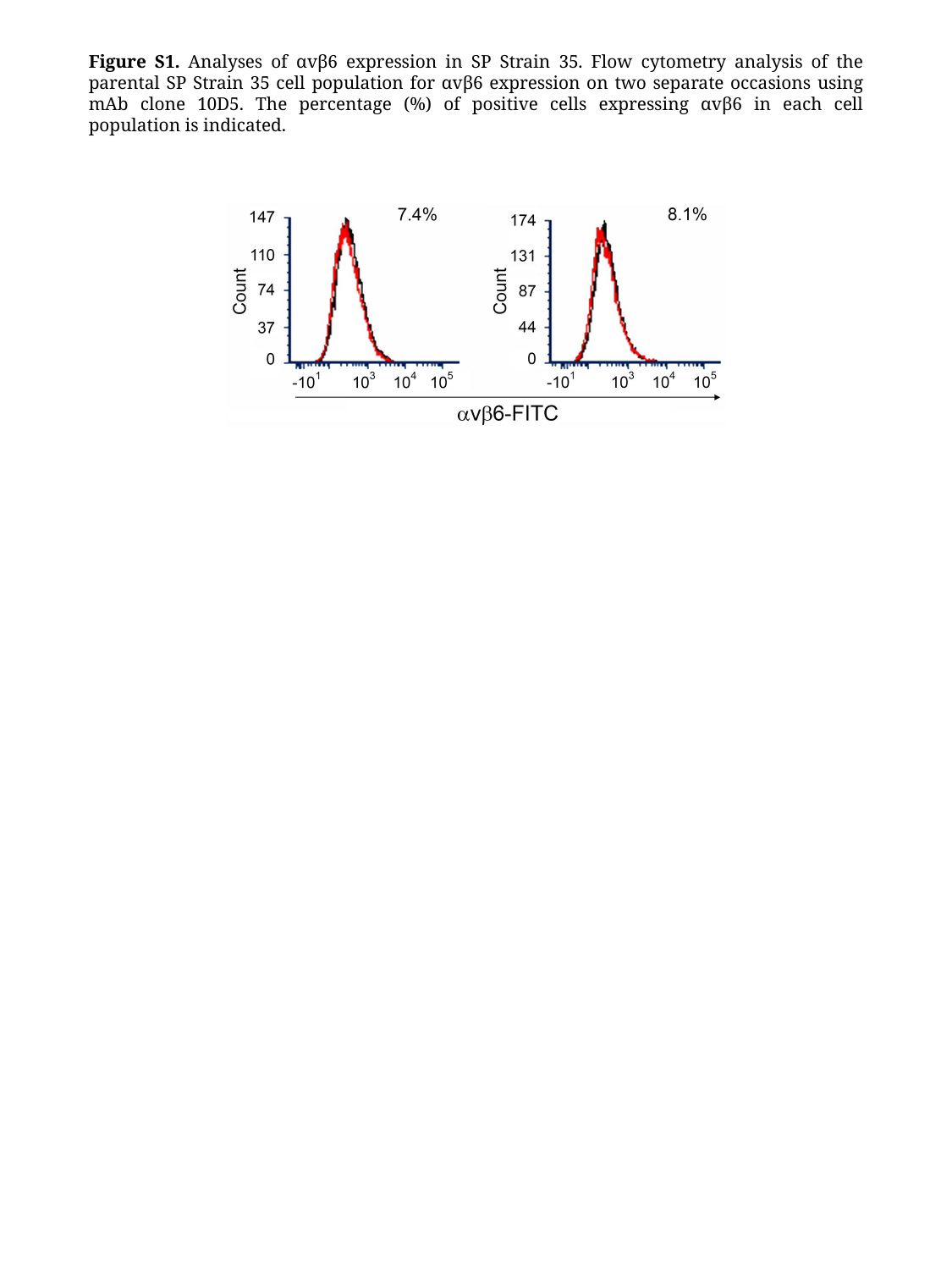

Figure S1. Analyses of αvβ6 expression in SP Strain 35. Flow cytometry analysis of the parental SP Strain 35 cell population for αvβ6 expression on two separate occasions using mAb clone 10D5. The percentage (%) of positive cells expressing αvβ6 in each cell population is indicated.

## Slide 2
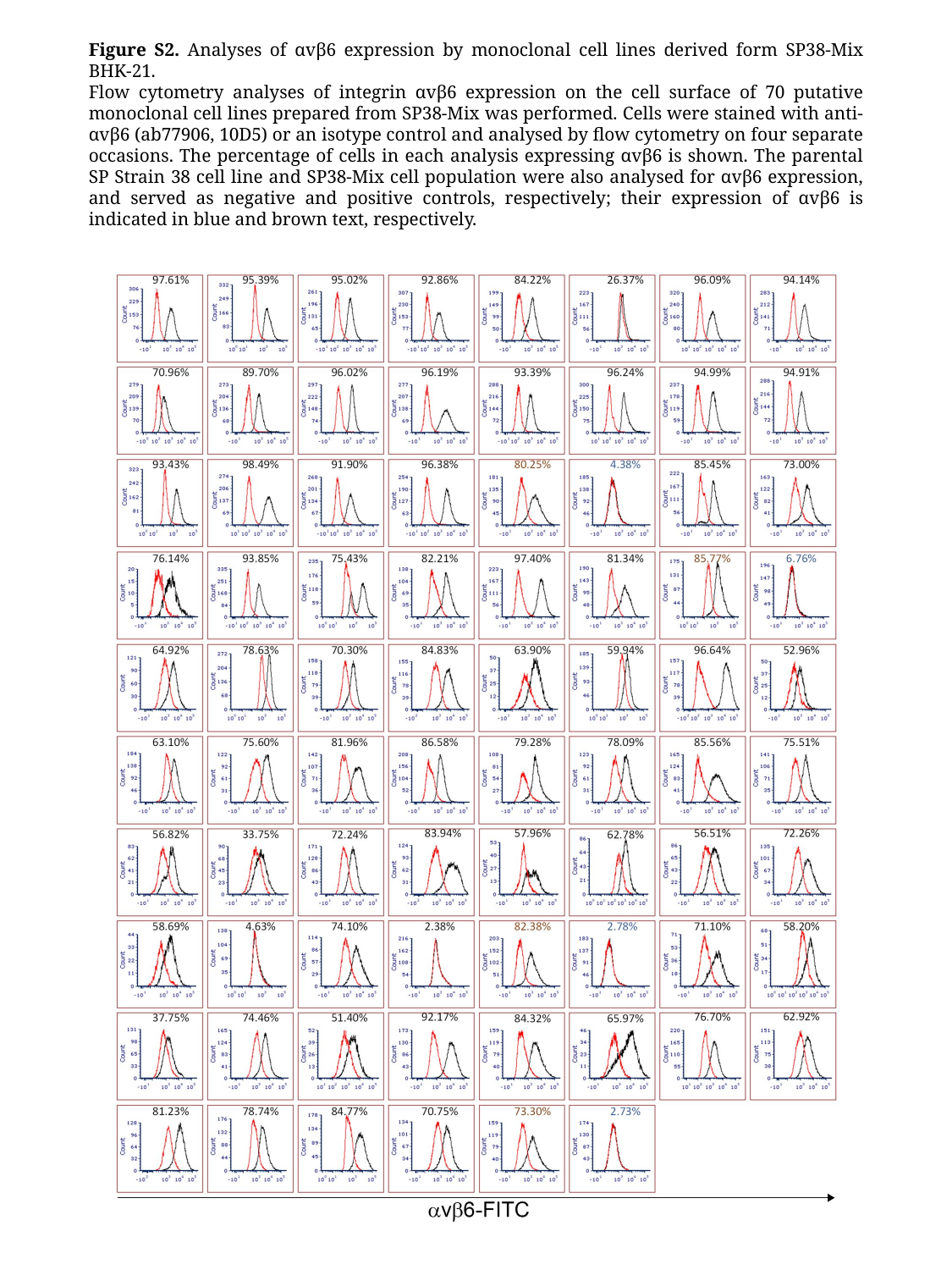

Figure S2. Analyses of αvβ6 expression by monoclonal cell lines derived form SP38-Mix BHK-21.
Flow cytometry analyses of integrin αvβ6 expression on the cell surface of 70 putative monoclonal cell lines prepared from SP38-Mix was performed. Cells were stained with anti-αvβ6 (ab77906, 10D5) or an isotype control and analysed by flow cytometry on four separate occasions. The percentage of cells in each analysis expressing αvβ6 is shown. The parental SP Strain 38 cell line and SP38-Mix cell population were also analysed for αvβ6 expression, and served as negative and positive controls, respectively; their expression of αvβ6 is indicated in blue and brown text, respectively.

## Slide 3
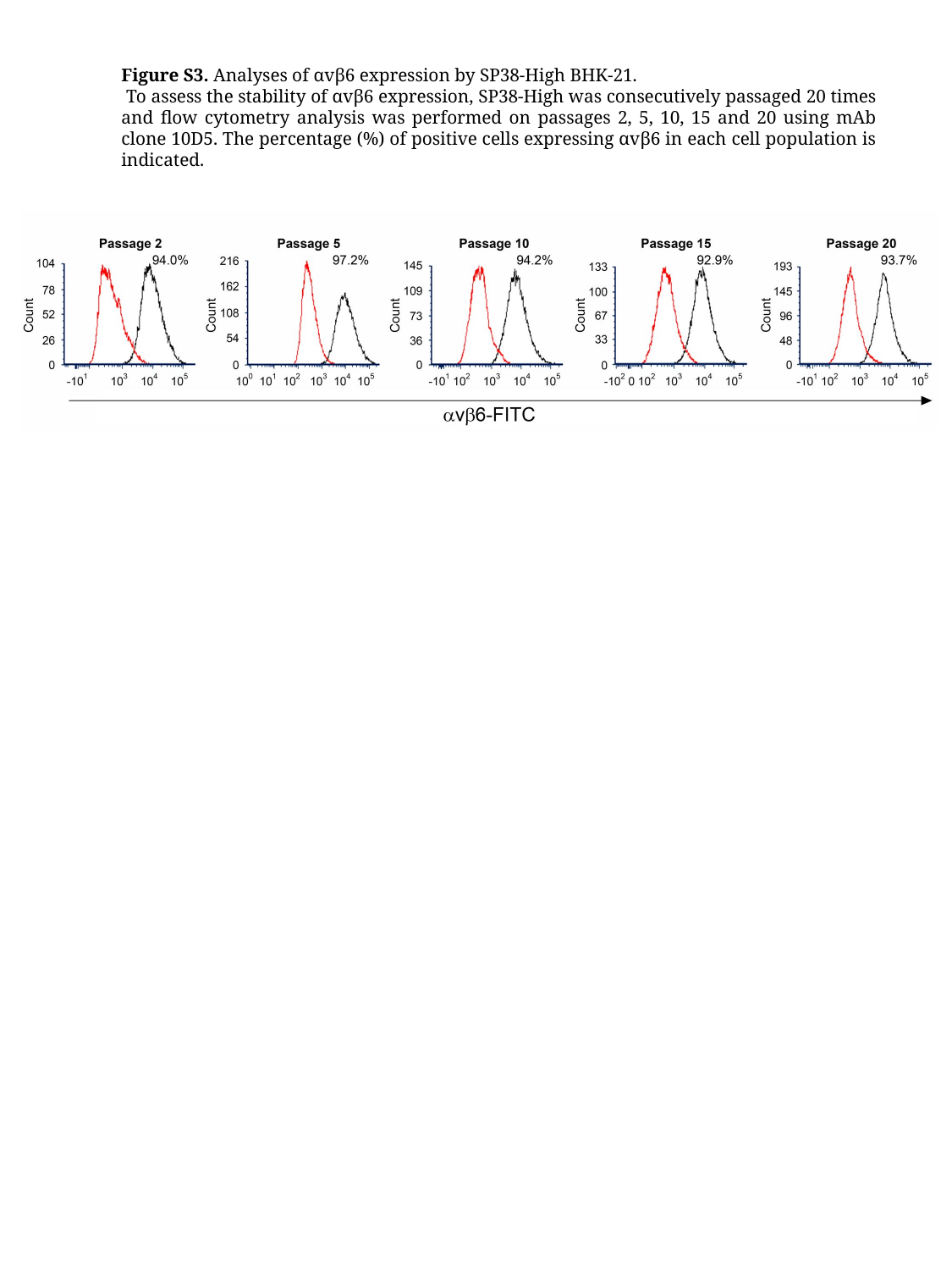

Figure S3. Analyses of αvβ6 expression by SP38-High BHK-21.
 To assess the stability of αvβ6 expression, SP38-High was consecutively passaged 20 times and flow cytometry analysis was performed on passages 2, 5, 10, 15 and 20 using mAb clone 10D5. The percentage (%) of positive cells expressing αvβ6 in each cell population is indicated.

## Slide 4
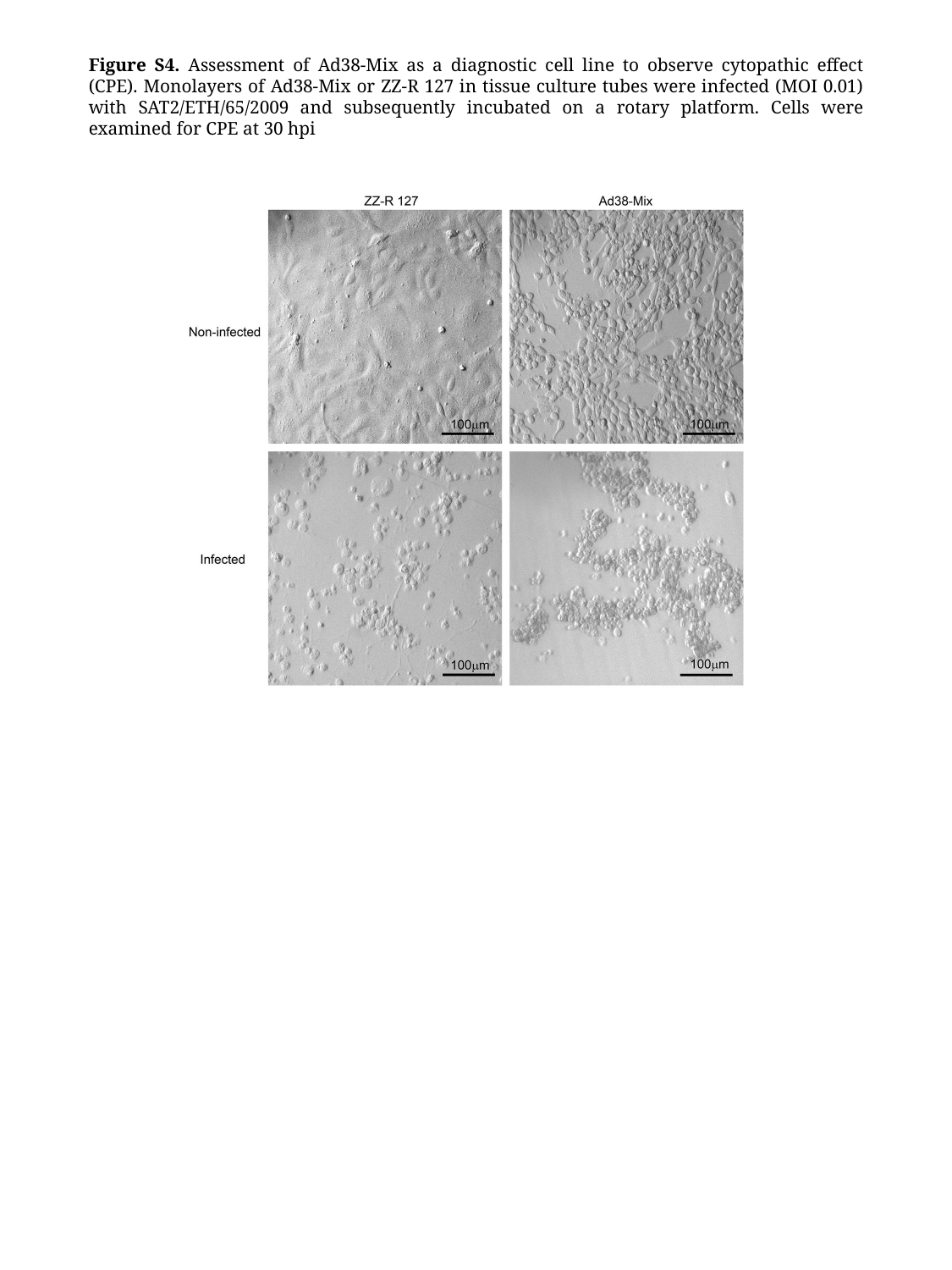

Figure S4. Assessment of Ad38-Mix as a diagnostic cell line to observe cytopathic effect (CPE). Monolayers of Ad38-Mix or ZZ-R 127 in tissue culture tubes were infected (MOI 0.01) with SAT2/ETH/65/2009 and subsequently incubated on a rotary platform. Cells were examined for CPE at 30 hpi

## Slide 5
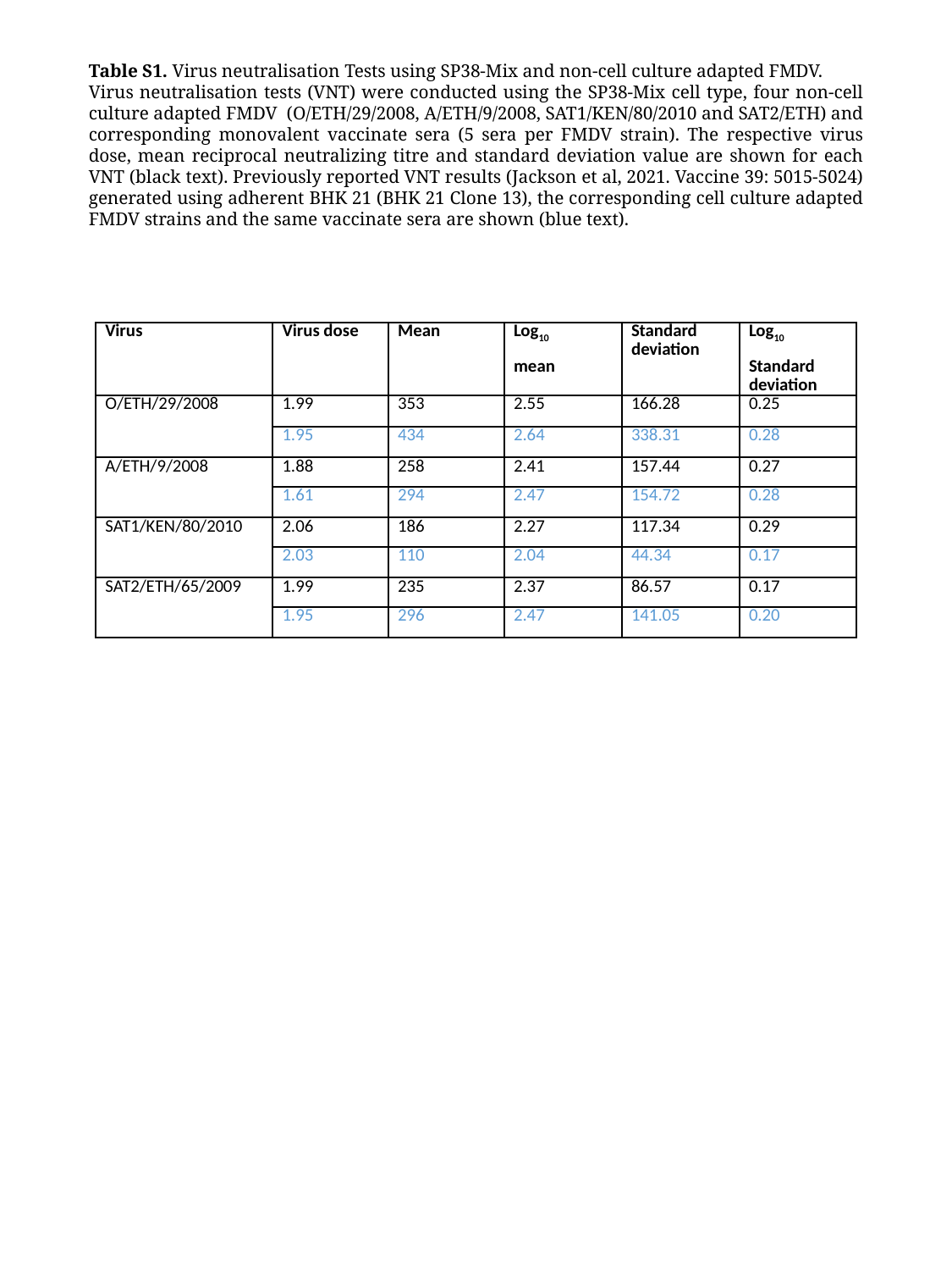

Table S1. Virus neutralisation Tests using SP38-Mix and non-cell culture adapted FMDV.
Virus neutralisation tests (VNT) were conducted using the SP38-Mix cell type, four non-cell culture adapted FMDV (O/ETH/29/2008, A/ETH/9/2008, SAT1/KEN/80/2010 and SAT2/ETH) and corresponding monovalent vaccinate sera (5 sera per FMDV strain). The respective virus dose, mean reciprocal neutralizing titre and standard deviation value are shown for each VNT (black text). Previously reported VNT results (Jackson et al, 2021. Vaccine 39: 5015-5024) generated using adherent BHK 21 (BHK 21 Clone 13), the corresponding cell culture adapted FMDV strains and the same vaccinate sera are shown (blue text).
| Virus | Virus dose | Mean | Log10 mean | Standard deviation | Log10 Standard deviation |
| --- | --- | --- | --- | --- | --- |
| O/ETH/29/2008 | 1.99 | 353 | 2.55 | 166.28 | 0.25 |
| | 1.95 | 434 | 2.64 | 338.31 | 0.28 |
| A/ETH/9/2008 | 1.88 | 258 | 2.41 | 157.44 | 0.27 |
| | 1.61 | 294 | 2.47 | 154.72 | 0.28 |
| SAT1/KEN/80/2010 | 2.06 | 186 | 2.27 | 117.34 | 0.29 |
| | 2.03 | 110 | 2.04 | 44.34 | 0.17 |
| SAT2/ETH/65/2009 | 1.99 | 235 | 2.37 | 86.57 | 0.17 |
| | 1.95 | 296 | 2.47 | 141.05 | 0.20 |
